# Harnessing generalized structural equation modelling to understand the pathway linking maternal and child factors to fruit and vegetable intake trajectories from toddlerhood to adolescence

**DOI:** 10.1007/s00394-025-03883-8

**Published:** 2026-01-24

**Authors:** Miaobing Zheng, Jia Ying Toh, Mya Thway Tint, Geeta Appannah, Wei Wei Pang, Keith M. Godfrey, Yap-Seng Chong, Yung Seng Lee, Fabian Yap, Johan G. Eriksson, Mary F. F. Chong

**Affiliations:** 1 School of Health Sciences, Faculty of Medicine and Health, University of New South Wales, Kensington, NSW 2033 Australia; 2 Institute for Human Development and Potential (IHDP), Agency for Science, Technology and Research (A*STAR), Singapore, Singapore; 3 Human Potential Translational Research Program, Yong Loo Lin School of Medicine, National University of Singapore, Singapore, Singapore; 4 Department of Nutrition, Faculty of Medicine and Health Sciences, Universiti Putra Malaysia, UPM Serdang, Selangor, 43400 Malaysia; 5 Division of Nutrition, Dietetics and Food Science, School of Health Sciences, IMU University, Kuala Lumpur , Malaysia; 6 Department of Obstetrics & Gynaecology, Yong Loo Lin School of Medicine, National University of Singapore, Singapore, Singapore; 7 Global Center for Asian Women’s Health, Yong Loo Lin School of Medicine, National University of Singapore, Singapore, Singapore; 8 Bia-Echo Asia Centre for Reproductive Longevity and Equality, Yong Loo Lin School of Medicine, National University of Singapore, Singapore, Singapore; 9 Medical Research Council Lifecourse Epidemiology Centre and National Institute for Health Research Southampton Biomedical Research Centre, University of Southampton and University Hospital, Southampton National Health Service Foundation Trust, Southampton, UK; 10 Department of Paediatrics, Yong Loo Lin School of Medicine, National University of Singapore, Singapore, Singapore; 11 Division of Paediatric Endocrinology and Diabetes, Khoo Teck Puat- National University Children’s Medical Institute, National University Hospital, National University Health System, Singapore, Singapore; 12 Duke-NUS Medical School, Singapore, Singapore; 13 Department of Paediatric Endocrinology, KK Women’s and Children’s Hospital, Singapore, Singapore; 14 Lee Kong Chian School of Medicine, Nanyang Technological University, Singapore, Singapore; 15 Department of General Practice and Primary Health Care, University of Helsinki and Helsinki University Hospital, Helsinki, Finland; 16 Saw Swee Hock School of Public Health, National University of Singapore and National University Health System, Singapore, Singapore

**Keywords:** Fruit and vegetable intakes, Dietary trajectories, Longitudinal modelling, Maternal and child factors, Maternal ethnicity

## Abstract

**Purpose:**

To identify longitudinal trajectories of fruit and vegetable intakes from toddlerhood to adolescence and examine integrative pathways with maternal and child factors in a multi-ethnic Asian cohort.

**Method:**

Data of 817 mother-child dyads from the GUSTO Study was used. Group-based trajectory modelling examined intake trajectories of fruits and vegetables from ages 18months to 12years. Generalized structural equation modelling evaluated the integrative direct and mediational pathways linking maternal and child factors to fruit and vegetable intake trajectories.

**Results:**

Two fruit intake trajectories: “High stable”(83.2%), “Low stable to decreasing”(16.8%) and three vegetable intake trajectories: “Consistently high”(78.8%), “Low to stable”(9.2%), “High decreasing”(12.0%) were identified. Malay ethnicity (vs. Indian) and boys (vs. girls) were directly and significantly associated with higher odds of adhering to the suboptimal “Low stable to decreasing” fruit intake (ORs 1.87 and 2.32) or “Low to stable/High decreasing” vegetable intake (ORs 3.49 and 1.80) trajectories, respectively. Every unit increase in maternal pregnancy diet quality score was significantly associated with 3–4% lower odds of following the suboptimal fruit or vegetable intake trajectory. Significant direct inverse associations were also observed between breastfeeding duration ≥ 6months (vs. <6months) or maternal university education and lower odds of following the suboptimal fruit or vegetable intake trajectory. Pregnancy diet quality partially mediated the association between maternal ethnicity and suboptimal fruit or vegetable intake trajectory.

**Conclusion:**

Interventions to promote child fruit and vegetable intakes should begin preconception or during pregnancy, prioritise Malay mothers, those with lower education, families with boys, and improve maternal diet quality while promoting breastfeeding.

**Supplementary Information:**

The online version contains supplementary material available at 10.1007/s00394-025-03883-8.

## Introduction

As rich sources of essential vitamins, minerals, dietary fibre, and antioxidants, fruits and vegetables are essential components of a healthy diet to maintain good health and well-being. Low fruit and vegetable intakes have been associated with an elevated risk of chronic diseases such as obesity, heart disease and certain cancers [1, 2]. Estimates from the World Health Organization (WHO) in 2017 showed that 3.9 million deaths globally were attributable to low fruit and vegetable intakes [3]. Despite the emphasis on increasing fruit and vegetable intake, low fruit and vegetable intake remains a global concern, affecting many countries worldwide [4–6].

Compelling evidence has shown food preferences and dietary habits are established early in life and are hard to change once established [7], highlighting early life as a critical window for initiation of dietary interventions. The developmental origins of health and disease (DOHaD) concept also cements the importance of early life exposures in programming later health behaviours [8, 9]. Several maternal and child factors such as maternal ethnicity, education, and dietary intakes and child sex were shown to be associated with fruit and vegetable intakes in children and adolescents [10]. However, existing evidence largely stems from cross-sectional associations with a paucity of research on factors influencing longitudinal trajectories of fruit and vegetable intakes [10].

Investigating determinants of dietary trajectories can uncover key factors influencing dietary changes over time, identify the critical time points when relationships start to emerge, and characterize at risk populations with suboptimal dietary trajectories. Such insights will inform when and with whom to initiate dietary interventions. Additionally, emerging studies primarily assessed trajectories of dietary quality or patterns and their influencing factors with very few investigating trajectories of food group intakes [11]. Studying changes in different components of diet (i.e., food groups) is fundamental to inform tailored interventions and guidelines specific to food groups such as fruit and vegetables [11].

Current literature typically uses multivariable regression to examine the independent associations of prenatal or postnatal factors with fruit and vegetable intakes, without considering the interplay between factors [9]. In this study, we utilize generalized structural equation modelling (GSEM) to delineate the respective sequential pathways linking maternal and child factors and fruit and vegetable intake trajectories. Unlike traditional regression, GSEM allows us to simultaneously model integrative pathways, including mediation, providing insights into how maternal and child factors jointly influence these trajectories and their relative contributions. In an Asian multi-ethnic cohort, the current study aimed to (1) describe intake trajectories of fruits and vegetables from toddlerhood to adolescence and (2) apply GSEM to identify maternal and child determinants of fruit and vegetable intake trajectories and quantify their relative contributions via direct and indirect pathways.

## Methods

### Study design and participants

Data from the Growing Up in Singapore Towards healthy Outcomes (GUSTO) cohort was used. The GUSTO cohort study is an on-going mother-offspring cohort aimed at evaluating the roles of pregnancy and early life factors including genetics, environmental and lifestyle factors in influencing child growth and development, and long-term programming of health and disease [12]. At study baseline, 1450 pregnant women aged 18 years and over who were Singapore citizens or permanent residents of Chinese, Malay or Indian ethnicity were recruited at their first trimester antenatal appointment from two major public maternity units in Singapore (National University Hospital and KK Women’s and Children’s Hospital) between June 2009 and September 2010. Informed written consent was obtained from each participant. Ethical approval was obtained from the SingHealth Centralized Institutional Review Board and National Healthcare Group Domain Specific Review Board (CIRB 2018/2767/D; DSRB D/2009/00021 and B/2014/00406). The detailed study protocol has been published [12]. The current analyses used data from mother-child dyads who participated in follow-up assessments at ages 18 months, 5, 7 and 12 years.

## Assessment of fruits and vegetable intakes

Child dietary intake was assessed at ages 18 months, 5, 7 and 12 years using quantitative food frequency questionnaires (FFQs). Development and validation of the FFQs have been reported elsewhere [13–15]. FFQs were completed by mothers from ages 18 months to 7 years, and by children at age 12 years. The FFQs captured frequency of food intake in the past month. Notably, the FFQ items were age-specific, with more food items being administered at ages 5 and 7 years than age 18 months. However, an abbreviated FFQ was administered at age 12 years to ease child participant burden. This variation limits direct comparability of absolute intake levels across ages but allows identification of relative trajectory patterns. Mothers or children reported the portion sizes of each food item using household measurements or standard cups, spoons and plates used in the Singapore setting. Frequency of intake per month was converted into daily equivalents. Intakes of each food item (grams per day) were obtained as daily equivalents multiplied by portion size reported by mothers or children [16]. For the current analysis, fruit intake was calculated as the daily intake of all fresh and dried fruit items. Vegetable intake was calculated as daily intakes of all vegetable items, excluding potato. Detailed fruit and vegetable items being captured over four time points are presented in Supplementary Table 1.

## Assessment of maternal and child factors

At 26–28 weeks gestation (third pregnancy visit), maternal age (in years), smoking status (current smoker/past smoker vs. non-smoker), ethnicity (Chinese vs. Malay vs. Indian), education (secondary and lower vs. post-secondary vs. university) and household income (Singapore dollars) were ascertained using questionnaires. Maternal height was measured to the nearest 0.1 cm using a Seca 213 Portable Stadiometer (SECA, Hamburg, Germany). Weight was measured to the nearest 0.1 kg using a calibrated electronic weighing scale (SECA 803: SECA, Hamburg, Germany). Measurements were performed in duplicates and the average of readings were used. Maternal dietary intake during pregnancy was assessed using a 24-hour recall administered by trained staff [17]. Overall diet quality was assessed using the Healthy Eating Index for pregnant women in Singapore (HEI-SGP) [18]. HEI-SGP has a score range of 0 to 100 that evaluates adequacy and quality of food groups and adherence to antenatal supplement recommendations with higher score indicating a better diet quality. Breastfeeding duration (≥ 6 versus < 6 months) was estimated using infant feeding questionnaires administered when infants were 3, 6, 9, and 12 months of age, where breastfeeding was defined as any consumption of breastmilk regardless of what else was consumed by the infants [19].

### Statistical analysis

Group based trajectory modelling (GBTM) [20, 21] was conducted to identify distinct trajectories of fruit and vegetable intakes (grams/day, log-transformed) at ages 18 months, 5, 7 and 12 years. GBTM identifies heterogenous groups within the sample that follow distinct trajectories [20, 21]. Censored normal distribution was assumed specifying linear, quadratic, and cubic functions of child age in months as independent variables and repeated measurements of fruit or vegetable intakes as the outcome variable. The polynomic function of age allows a non-linear trajectory in each group. Models with two to five groups were conducted. Fruit and vegetable intakes were log-transformed before GBTM analyses to account for non-normal distribution and varying number of fruit and vegetable FFQ items being used to capture intakes at various time points. Selection of final number of trajectory groups was based on several criteria: Bayesian Information Criteria (BIC), model parsimony (simpler models), proportion of individuals in each group (> 5%), entropy (a measure of classification accuracy, range 0–1, higher values indicate better separation), and interpretability. Larger BIC (less negative) and entropy indicate a better model fit [20, 21].

Descriptive analysis was conducted to summarize sample characteristics by identified fruit and vegetable intake trajectory groups. Associations between maternal and child factors and identified fruit and vegetable intake trajectories were examined using generalized structural equation modelling (GSEM) [22]. GSEM examines direct associations between various factors and the outcome of interest by estimating multiple pathways simultaneously and allows estimation of total and indirect (mediational) pathways. By comparing the strength of the path coefficients between factors and outcome of interest, relative contributions of factors can be determined. Separate GSEM with a maximum likelihood estimation was conducted for fruit and vegetable intake trajectory groups, respectively. Continuous variables (maternal pregnancy BMI, maternal pregnancy diet quality, child birth weight) were analysed using Gaussian family with identity link, whereas categorical variables (maternal ethnicity, education, smoking, breastfeeding duration, child sex, fruit/vegetable trajectory groups) were analysed using binomial family with logit link. For ease of interpretation, maternal ethnicity was coded into two dummy variables representing ‘Malay’ versus ‘Chinese’ and ‘Indian’ versus ‘Chinese’. Likewise, maternal education was coded into ‘university education’ versus ‘secondary education or lower’ and ‘post-secondary education’ versus ‘secondary education or lower’. Household income was not examined as a determinant as it was highly correlated with maternal education (*r* = 0.60) and had higher missingness than maternal education (8% vs. 3%).

Various GSEMs were constructed by including all possible pathways (or associations) based on theoretical plausibility and prior literature. Model selection was based on comparison of model fit statistics (i.e., likelihood ratio test, and Akaike and Bayesian information criteria) and clinical interpretability. Maternal age and child birth weight showed no significant association with fruit and vegetable intake trajectories and removal of both improved the model fit (Likelihood ratio test: χ^2^(11) = 173.40, *P* < 0.001 for fruit; χ^2^(11) = 174.58, *P* < 0.001 for vegetable). Hence child birth weight was excluded in the final GSEM model. The best fitting model included the following pathways (exposure ⟶outcome):

- Child sex, maternal ethnicity, maternal education, maternal pregnancy diet quality, pregnancy BMI, pregnancy smoking status, breastfeeding duration ⟶ Low fruit or vegetable intake trajectory group.
- Maternal ethnicity ⟶ Maternal education.
- Maternal ethnicity, maternal education ⟶Maternal pregnancy BMI.
- Maternal ethnicity, maternal education, maternal pregnancy smoking ⟶ Maternal pregnancy diet quality.
- Maternal ethnicity, maternal education ⟶ Maternal pregnancy smoking.
- Maternal education, maternal pregnancy BMI, maternal diet quality, maternal pregnancy smoking ⟶Breastfeeding duration.

The “program” command was used to conduct nonparametric bootstrapping of 1000 replicates to obtain bias-corrected and normal-based confidence intervals for all pathways. The indirect (mediational) effect was obtained by the product method (calculated as the product of exposure-mediator *(a)* and mediator-outcome *(b)* path coefficients) and total effect was calculated as the sum of direct and indirect effect [23]. Percentage mediation was estimated as indirect effect divided by total effect. All analyses were conducted in Stata 18 (StataCorpLLC) with statistical significance set at *P* < 0.05 (two-sided).

## Results

Children with two or more dietary intake measurements over four time points were included in the analyses to identify fruits and vegetable intake trajectories (*n* = 817). Children with missing data on child and maternal factors (*n* = 136) were excluded, resulting in 681 children being included in the GSEM analysis (Supplementary Fig. 1). Comparison of sample characteristics between those included and excluded from the analysis showed that excluded participants had lower birth weight (mean difference: -0.17 kg, *p* < 0.001), younger mothers (mean difference: -1.29 years, *p* < 0.001), lower maternal pregnancy diet quality (mean difference: -2.04 scores, *p* = 0.015), lower proportion of mothers with Indian ethnicity (15.6% vs. 21.7%, *p* = 0.008) and university education (30.4% vs. 35.1%, *p* = 0.023). However, child sex, maternal pregnancy BMI, pregnancy smoking status and breastfeeding duration did not differ between those included and excluded from the current analyses (Supplementary Table 2).

### Fruit and vegetable intake trajectories

For fruit intake, relative to the model with 2 groups, models with 3–5 groups showed better BIC and entropy, but one of the groups had < 5% of the sample. Thus, the model with 2 groups (BIC: -4500.4, entropy: 0.89) with good interpretability was chosen for fruit intake. For vegetable intake, the model with 3 groups was selected as it had relatively good BIC (-4878.6), entropy (0.88), and interpretability compared to other models with 2, 4 and 5 groups (Supplementary Table 3).

**Fig. 1:**
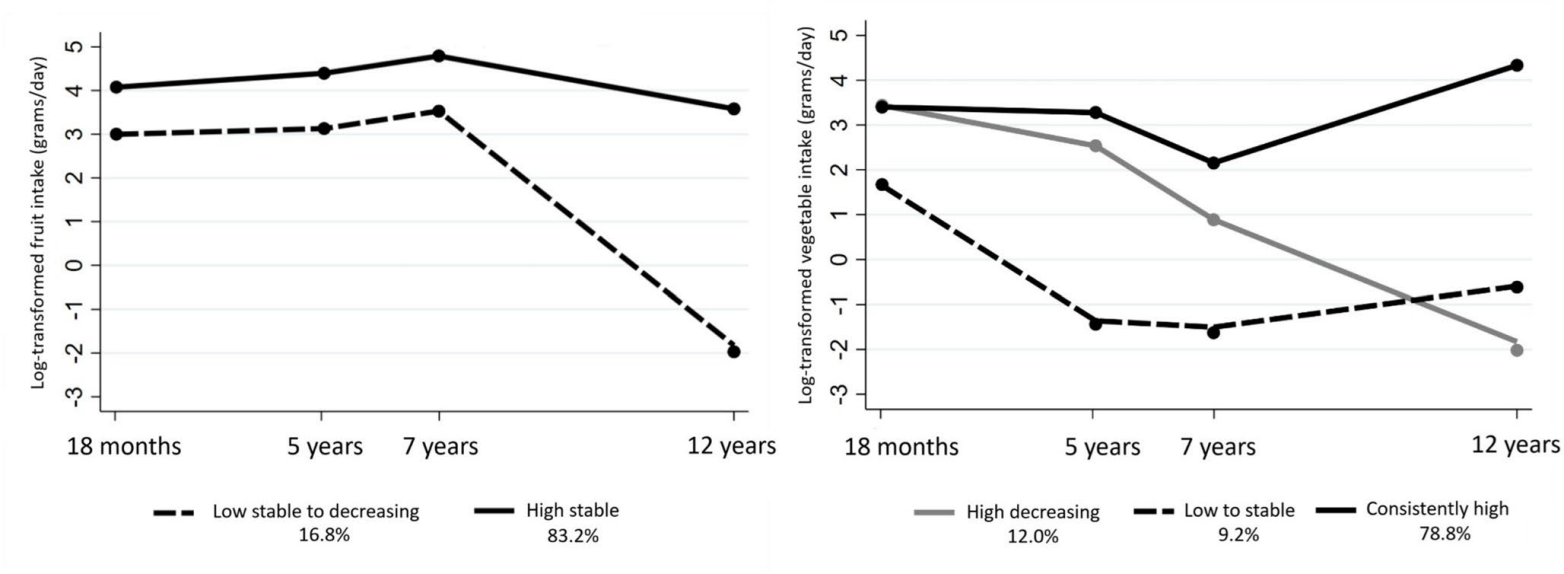
Fruit and vegetable intake (log-transformed) trajectories from ages 18 months to 12 years the GUSTO cohort

Fruit and vegetable intake trajectory groups from ages 18 months to 12 years are illustrated in Fig. 1. For fruit intake trajectories, most children (83.2%) followed a “High stable” intake trajectory with fairly stable intakes from age 18 months to 12 years. The remaining children (16.8%) had stable intakes from ages 18 months to age 7 years followed by a sharp decline until age 12 years. As it had lower intakes of fruit over time than the other group, it was named as the ‘Low stable to decreasing’ fruit intake trajectory group (Fig. 1).

Two of the three vegetable intake trajectory groups had high baseline intakes at age 18 months. Of these, one group (78.8%) exhibited consistently high intakes across all time points, with a slight dip from ages 5 to 7 years, and then an increase until age 12 years. In contrast, the other group (12.0%) started with high baseline intakes but continued with a sharp decline in intake subsequently, until age 12 years. The two groups are named as the “Consistently high” and “High decreasing” vegetable intake groups, respectively. The third group (9.2% of children) showed low baseline intakes, followed by a decline until age 5 years, but intake stabilized until age 12 years and is referred as the “Low to stable” vegetable intake trajectory group (Fig. 1).

## Sample characteristics by identified fruit and vegetable intake trajectory groups

Comparison of sample characteristics by fruit intake trajectory groups is shown in Table 1. Compared to the ‘High stable’ fruit intake trajectory, the ‘Low stable to decreasing’ fruit intake trajectory group had a lower proportion of girls and children who were breastfed for more than 6 months. Moreover, the ‘Low stable to decreasing’ fruit intake group had a higher proportion of mothers who were smokers, of Malay ethnicity, below university education, lower diet quality during pregnancy, but higher pregnancy BMI than the ‘High stable’ group (all *P* < 0.05). No evidence of significant differences was found in birth weight or maternal age according to the two fruit intake trajectory groups.

**Table 1:** Sample characteristics according to fruit and vegetable intake trajectory groups

	Fruit intake trajectory groups					Vegetable intake trajectory groups						
	Low stable to decreasing (*n* = 137)		High stable (*n* = 676)			High decreasing (*n* = 98)		Low to stable (*n* = 75)		Consistently high (*n* = 644)		
	n	% or mean(SD)	n	% or mean(SD)	*P*-value	n	% or mean(SD)	n	% or mean(SD)	n	% or mean(SD)	*P*-value
Child sex					< 0.001							0.005
Girls	45	32.8%	343	50.7%		34	34.7%	30	40.0%	324	50.6%	
Boys	92	67.2%	333	49.3%		64	65.3%	45	60.0%	316	49.4%	
Child birth weight (kg)	137	3.09(0.49)	676	3.10(0.47)	0.915	98	3.08(0.49)	75	3.10(0.45)	640	3.10(0.48)	0.951
Maternal age (years)	134	30.85(5.39)	657	31.47(5.04)	0.194	97	30.99(5.59)	71	29.69(5.79)	623	31.62(4.91)	0.008
Maternal BMI (kg/m^2^)	131	27.06(5.11)	642	26.01(4.29)	0.014	95	27.06(5.00)	68	27.47(5.18)	610	25.91(4.24)	0.003
Maternal smoking					0.003							< 0.001
Non-smoker	107	79.9%	585	89.2%		81	83.5%	50	70.4%	561	90.2%	
Ex- or current-smoker	27	20.1%	71	10.8%		16	16.5%	21	29.6%	61	9.8%	
Maternal ethnicity					< 0.001							< 0.001
Chinese	61	44.5%	415	61.9%		47	48.5%	14	18.9%	415	65.2%	
Malay	61	44.5%	144	21.5%		41	42.3%	51	68.9%	113	17.8%	
Indian	15	11.0%	111	16.6%		9	9.3%	9	12.2%	108	17.0%	
Maternal education					0.001							< 0.001
Secondary/lower	52	38.5%	181	27.4%		39	40.2%	40	55.6%	154	24.6%	
Post-secondary	54	40.0%	229	34.7%		41	42.3%	23	31.9%	219	35.0%	
University	29	21.5%	250	37.9%		17	17.5%	9	12.5%	253	40.4%	
Maternal pregnancy diet quality score	125	49.11(12.92)	604	53.30(13.81)	0.002	92	48.08(12.80)	65	47.65(13.50)	572	53.87(13.66)	< 0.001
Breastfeeding duration					< 0.001							< 0.001
< 6months	99	75.6%	363	56.4%		67	73.6%	56	82.4%	339	55.0%	
≥ 6months	32	24.4%	281	43.6%		24	26.4%	12	17.7%	277	45.0%	

BMI, body mass index; SD, standard deviation

Results for the vegetable intake trajectory groups were largely similar for most factors apart from maternal age and ethnicity. Relative to the ‘Consistently high’ vegetable intake trajectory group, the ‘High decreasing’ and ‘Low to stable’ groups had a lower proportion of girls, children whose mothers were non-smokers and had university education. Mothers of children from the ‘High decreasing’ and ‘Low to stable’ groups also had higher BMI but lower diet quality during pregnancy than the ‘Consistently high’ group (*P* < 0.05). ‘Low to stable’ group had mothers who were slightly younger than the ‘High decreasing’ and ‘Consistently high’ groups (*P* < 0.05). For maternal ethnicity, the ‘Low to stable’ group and the ‘Consistently high’ group had about two third of children whose mothers were of Malay or Chinese ethnicity, respectively. In contrast, the ‘High decreasing’ group had a similar proportion of children with mothers of Chinese and Malay ethnicity. No between-group differences were found for birth weight (Table 1).

## Direct and indirect pathways linking maternal and child factors and suboptimal fruit intake trajectory

**Fig. 2:**
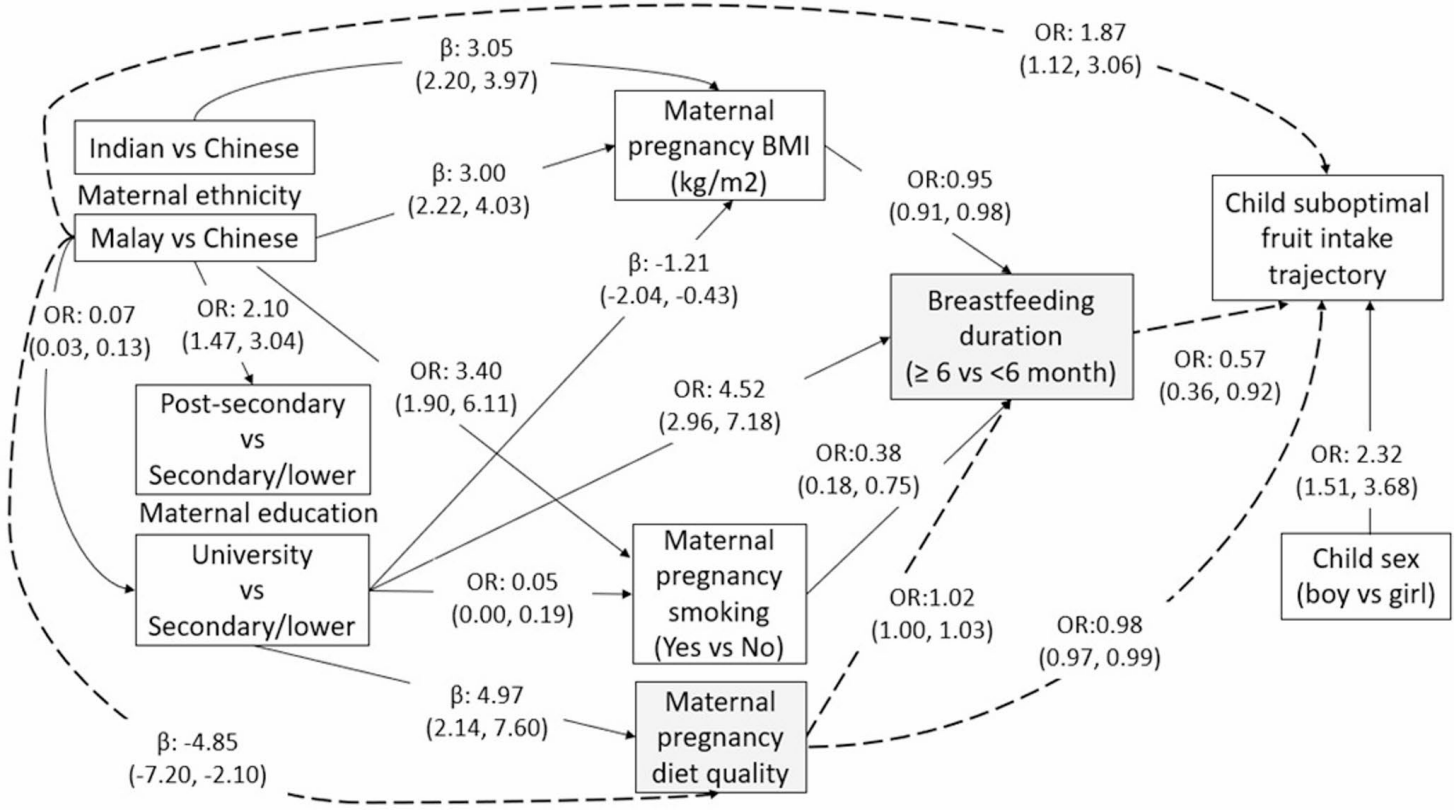
Pathways (direct associations) linking maternal ethnicity, education, pregnancy smoking, pregnancy body mass index (BMI), pregnancy diet quality, child sex, and the child suboptimal ‘Low stable to decreasing’ vs. ‘High stable’ fruit intake trajectory group. Effect sizes of all direct pathways are shown as β (beta-coefficient) and OR (odds ratio) with 95% confidence intervals. Dotted lines represent mediation with variables in grey boxes as mediators

Figure 2 illustrates direct associations between maternal and child factors and the ‘Low stable to decreasing’ versus ‘High stable’ fruit intake trajectory groups. Due to lower overall intake or declining patterns, ‘Low stable to decreasing’ is referred as the ‘suboptimal’ fruit intake group thereafter for simplicity and ease of interpretation. Child sex, maternal ethnicity, pregnancy diet quality score, and breastfeeding duration were directly associated with a child low fruit intake trajectory. Boys showed higher odds (OR 2.32; 95% CI 1.51, 3.68) of following the suboptimal fruit intake trajectory than girls. Children of mothers of Malay ethnicity, but not of Indian ethnicity, had higher odds of following the suboptimal fruit intake trajectory (OR 1.87; 95% CI 1.12, 3.06) than children of mothers of Chinese ethnicity. Every one-score increase in maternal pregnancy HEI-SGP (score range 0-100) was associated with lower odds (OR 0.98; 95% CI 0.97, 0.99) of children following the suboptimal fruit intake trajectory. Relative to children who were breastfed < 6months, children who were breastfed for ≥ 6months had lower odds (OR 0.57; 95% CI 0.36, 0.92) of following the suboptimal fruit intake trajectory. Neither maternal education, pregnancy BMI, nor pregnancy smoking was directly associated with the child suboptimal fruit intake trajectory. Detailed results for all direct pathways are presented in Supplementary Tables 4 and 5.

The relative contributions of maternal and child factors were assessed by comparing the strength (magnitude of effect size: beta-coefficients and ORs) of the direct pathways. Child sex was found to be the strongest determinant of suboptimal fruit intake trajectory. Maternal ethnicity was a pivotal determinant as it was implicated in multiple pathways leading to child suboptimal fruit intake trajectory. Apart from a significant direct association, maternal ethnicity was also indirectly associated with the child suboptimal fruit intake trajectory via the mediating effect of maternal pregnancy diet quality. The association between maternal ethnicity (Malay vs. Chinese) and higher odds of following the child suboptimal fruit intake trajectory was partially mediated by maternal pregnancy diet quality, contributing to 11% of total effect **(**Table 2**)**. Furthermore, maternal ethnicity also influenced multiple intermediate downstream factors including maternal education, BMI, and pregnancy smoking, all of which were linked to breastfeeding duration, which in turn was associated with the suboptimal fruit intake trajectory. In addition to the direct association, maternal pregnancy diet quality score was also indirectly associated with child suboptimal fruit intake trajectory via the mediating effect of breastfeeding duration. Breastfeeding duration (≥ 6 vs. < 6 months) mediated 36% of the total effect between maternal pregnancy diet quality and lower odds of following the child suboptimal fruit intake trajectory (Table 2).

**Table 2:** Mediational pathways between maternal and child factors and child suboptimal fruit (‘Low stable to decreasing’) or vegetable (‘High decreasing and low to Stable’) intake trajectories

	Effect size	95% confidence interval
*Maternal ethnicity (Malay vs. ref Chinese) and suboptimal fruit intake trajectory*		
Direct effect	0.63	(0.11, 1.12)
Indirect effect via maternal pregnancy diet quality	0.08	(0.01,0.18)
Total effect	0.70	(0.22,1.22)
% mediation by maternal pregnancy diet quality	11%	
*Maternal pregnancy diet quality and suboptimal fruit intake trajectory*		
Direct effect	− 0.016	(− 0.030, − 0.001)
Indirect effect via breastfeeding duration	− 0.009	(− 0.023, − 0.001)
Total effect	− 0.025	(− 0.043, − 0.007)
% mediation by breastfeeding duration	36%	
*Maternal ethnicity (Malay vs. ref Chinese) and suboptimal vegetable intake trajectory*		
Direct effect	1.25	(0.76, 1.72)
Indirect effect via maternal education	2.00	(0.31, 3.93)
Indirect effect via maternal pregnancy diet quality	0.11	(0.03, 0.23)
Total effect	3.36	(1.75, 5.18)
% mediation by maternal education	60%	
% mediation by maternal pregnancy diet quality	3%	
*Maternal education (University vs. ref Secondary and lower) and suboptimal vegetable intake trajectory*		
Direct effect	− 0.76	(− 1.42, − 0.12)
Indirect effect via maternal pregnancy diet quality	− 0.11	(− 0.24, − 0.03)
Total effect	− 0.87	(− 1.51, − 0.25)
% mediation by maternal pregnancy diet quality	13%	

Effect sizes are presented as log-odds; 95% confidence interval (upper, lower)

Reference category for suboptimal ‘Low stable to decreasing’ fruit intake is ‘High stable’

Reference category for suboptimal ‘High decreasing and Low to Stable’ vegetable intake is ‘Consistently high’

### Direct and indirect pathways linking maternal and child factors and suboptimal vegetable intake trajectory

**Fig. 3:**
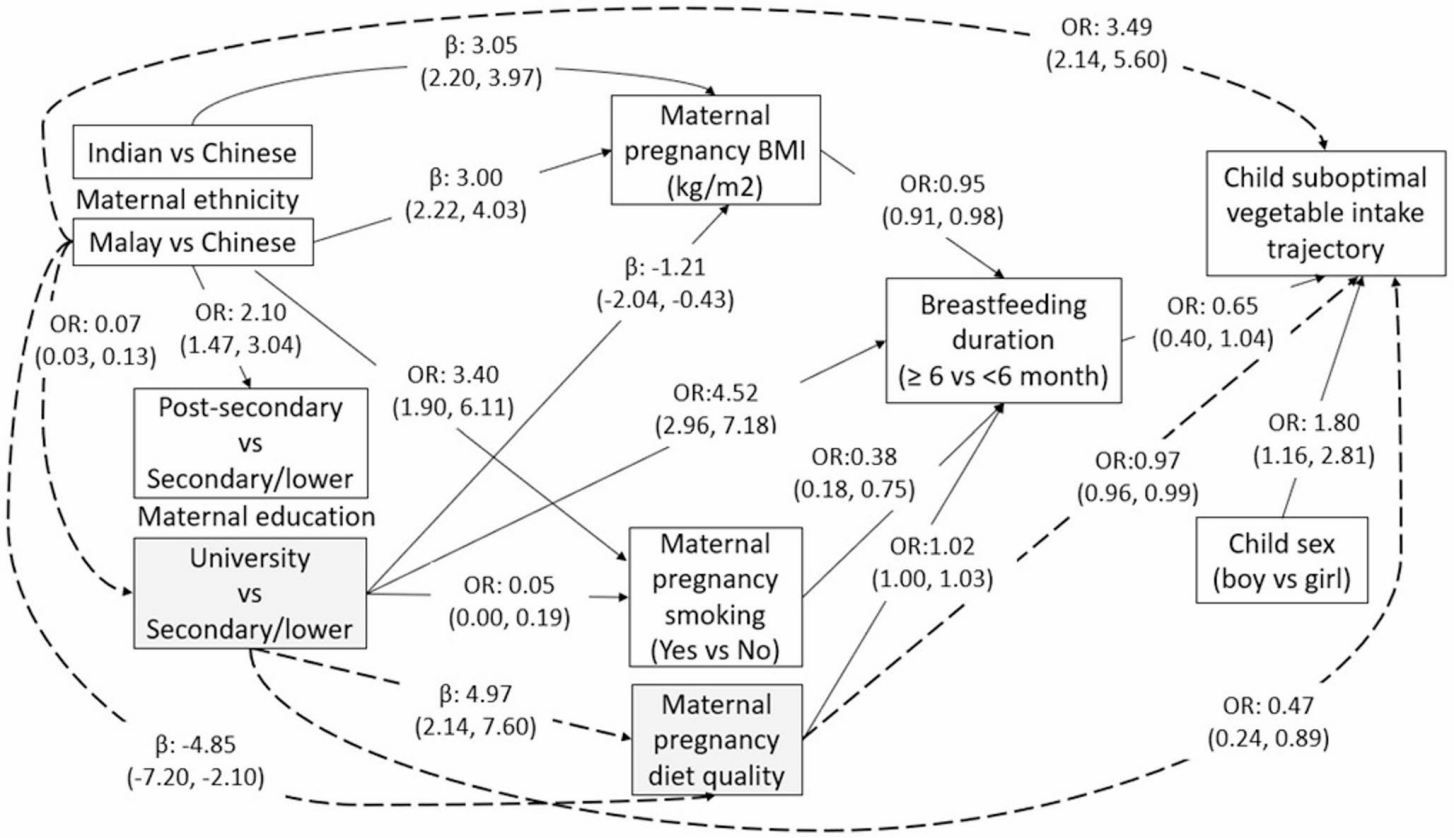
Pathways (direct associations) linking maternal ethnicity, education, pregnancy smoking, pregnancy body mass index (BMI), pregnancy diet quality, child sex, and the child suboptimal ‘High decreasing and Low to Stable’ vs. ‘Consistently high’ vegetable intake trajectory group. Effect sizes of all direct pathways are shown as β (beta-coefficient) and OR (odds ratio) with 95% confidence intervals. Dotted lines represent mediation with variables in grey boxes as mediators

Given the small proportions of the ‘High decreasing’ and ‘Low to stable’ vegetable intake trajectory groups and largely similar sample characteristics, they were combined and collectively referred as the suboptimal intake group and compared with the ‘Consistently high’ vegetable intake group in GSEM. With respect to determinants of child suboptimal vegetable intake trajectory group **(**Fig. 3**)**, results were largely similar to those of fruit intake trajectories, with child sex (boys vs. girls OR 1.80; 95% CI 1.16, 2.81), maternal ethnicity (Malay vs. Chinese OR 3.49; 95% CI 2.14, 5.60) and pregnancy diet quality score (OR 0.97; 95% CI 0.96, 0.99) being identified as significant determinants. However, a borderline significant direct association (OR 0.65; 95% CI 0.40, 1.04) was observed for breastfeeding duration (≥ 6 vs. < 6 months). Moreover, a significant direct association was found between maternal education and child suboptimal vegetable trajectory intake group. Children of mothers with university education (OR 0.47; 95% CI 0.24, 0.89), but not those with post-secondary education, had lower odds of following the suboptimal vegetable intake trajectory than children of mothers with secondary or lower education. No evidence of direct associations was found for maternal pregnancy BMI and smoking with the child suboptimal vegetable intake trajectory.

With respect to the suboptimal vegetable intake trajectory and the relative influences of maternal and child factors, maternal ethnicity was the most influential factor both in terms of the strength of the direct pathway and its impact on multiple intermediate factors. Maternal ethnicity showed the strongest direct association, followed by child sex, maternal education and pregnancy diet quality. Maternal ethnicity was also associated with suboptimal vegetable intake trajectory via a sequential pathway through maternal education followed by pregnancy diet quality, which subsequently led to suboptimal vegetable intake trajectory. The association between maternal ethnicity (Malay vs. Chinese) and higher odds of following the child suboptimal vegetable intake trajectory was mediated by maternal education (60% of total effect) and maternal pregnancy diet quality (3% of total effect) (Table 2). Maternal pregnancy diet quality score also mediated 13% of the total effect between maternal education (university versus secondary/lower) and lower odds of following the child suboptimal vegetable intake trajectory (Table 2).

## Discussion

In a longitudinal, multi-ethnic Asian cohort, distinct fruit and vegetable intake trajectories from toddlerhood to early adolescence were identified. Two fruit intake trajectories emerged: “High stable” and “Low stable to decreasing”. Three vegetable intake trajectories were identified: “Consistently high,” “High decreasing,” and “Low to stable.” Child sex, maternal ethnicity, and pregnancy diet quality were directly associated with suboptimal trajectories for fruit (“Low stable to decreasing”) and vegetable (“High decreasing/Low to stable”) intakes. A direct inverse association was observed for longer breastfeeding duration and the suboptimal child fruit intake trajectory, and for higher maternal education and the suboptimal child vegetable intake trajectory. Maternal ethnicity was the most influential determinant, having a direct association with suboptimal intake trajectories of fruit or vegetable while also exerting direct influences on multiple downstream factors (e.g., maternal education, pregnancy BMI, smoking status, diet quality) that in turn led to suboptimal intake trajectories of fruits and vegetables.

The present study is the first to examine respective fruit and vegetable intake trajectories from toddlerhood to adolescence. Our study revealed that distinct trajectories of fruit and vegetable intakes emerged from toddlerhood, making it unique from previous cohort studies in children which primarily examined changes in combined fruit and vegetable intake from late childhood (ages 8–10 years) until adolescence or early adulthood [24–27]. Of these, only one study examined intake trajectories, but it assessed joint trajectories with breakfast eating from childhood to adolescence [27]. Data from many countries have revealed a higher prevalence of inadequate vegetable intake than of fruit intake [4–6], highlighting the need to examine fruit and vegetable intakes separately. In line with our study findings, a UK study examining the changes in respective fruit and vegetable intakes from ages 2 to 23 years using four waves of national survey data also discovered a decline in fruit intake starting from age 7 years [28]. It is important to note that children from the ‘High stable’ or ‘Consistently high’ intake trajectory in our cohort consumed less fruits and vegetables than WHO or Singaporean dietary recommendations [3, 29], emphasizing the importance of promoting fruit and vegetable intakes from early life.

Expanding findings from previous studies that reported cross-sectional associations of maternal and child factors with fruit and vegetable intakes in childhood or adolescence [30–35], our study provides novel findings on the relative importance of these factors in influencing trajectories of fruit and vegetable intakes. No previous study has simultaneously assessed sequential direct and indirect pathways, providing valuable insights into the interplay of maternal and child factors and how they co-jointly influence child suboptimal fruit and vegetable intakes. Notably, maternal ethnicity was identified as the most pivotal distal factor influencing suboptimal fruit and vegetable intake trajectories as evidenced by a direct association as well as indirect associations via maternal education and/or pregnancy diet quality. Specifically, our mediation analyses showed that mothers of Malay ethnicity were more likely to have low maternal diet quality during pregnancy, which in turn led to increased risk of their child following the suboptimal fruit intake trajectory. Similar findings were found for vegetable intake trajectories, with maternal education being identified as an additional mediator underlying the association between maternal ethnicity and the suboptimal intake trajectory. Women with higher education are more likely to have better diet quality, which could promote positive parental modelling of healthy dietary intakes and home availability of healthy foods such as fruits and vegetables, resulting in better child dietary intakes [36]. In addition, maternal dietary intakes before and during pregnancy may affect child’s food preferences by influencing a child’s food flavour exposure in utero [37–39]. We also observed direct associations of maternal ethnicity with pregnancy diet quality, BMI and smoking, which were inversely linked with longer breastfeeding duration. Subsequently, longer breastfeeding duration decreased the risk of the child belonging to the suboptimal fruit and vegetable intake trajectories. Better diet quality, a healthy body weight status, and lower rate of smoking during pregnancy, as a proxy of a healthy lifestyle [40, 41], may promote good milk supply and promote longer duration of breastfeeding [42]. Breastfeeding may also shape children’s appetite and food preferences via exposing infants to flavours through breastmilk [43].

Another key determinant of suboptimal fruit and vegetable intake trajectories was child sex, with boys being more likely to follow a suboptimal fruit or vegetable intake trajectory than girls. Similar gender differences in fruit and vegetable intakes have been reported previously [30]. It is hypothesized that females may have different taste preference and tend to prefer fruits and vegetables more than males [44]. In addition, females are more likely to be impacted by external influences (i.e., parental, peers, and social) and more receptive to dietary advice or more health conscious as they grow older, resulting in healthier dietary intakes (e.g., higher fruit and vegetable intakes) at older ages [45].

Our study has several strengths including being the first to examine early determinants of longitudinal trajectories of fruit and vegetable intakes from toddlerhood to early adolescence in an Asian multi-ethnic cohort. The repeated measurements of fruit and vegetable intakes enabled the use of GBTM to elucidate dynamic changes in fruit and vegetable intakes over time. GBTM retains participants with partial data under missing-at-random assumptions, reducing attrition bias. The use of GSEM is another strength which allowed the simultaneous evaluation of the integrative pathways linking various maternal and child factors with fruit and vegetable intake trajectories, enabling quantification of both direct and indirect/mediational effects. GSEM also has potential to guide targeted interventions by identifying key factors that could be prioritized for intervention by assessing the relative contribution of various factors. The long duration of follow-up is another strength which enabled assessment of changes in fruits and vegetables from toddlerhood to early adolescence.

Limitations of our study include that varying numbers of fruit and vegetable items were captured by FFQs over four time points. Varying FFQ items and portion size estimation could introduce measurement error, and biomarker calibration was unavailable. We captured groups within the sample that had differential intake trajectories of fruit and vegetables over the four time points. While intake trajectories reflect relative patterns, absolute intake changes cannot be inferred. Additionally, dietary intake was self-reported by either mothers or children, and reporting bias cannot be dismissed. The excluded participants, compared with those included in the analysis, had characteristics associated with a higher likelihood of following the suboptimal fruit or vegetable intake trajectories (i.e., lower education, lower pregnancy diet quality, and shorter breastfeeding duration), potentially leading to underestimation of true effect sizes. Given the observational design of the study, casual inference cannot be drawn, and unmeasured/residual confounding is also possible. Lastly, mothers with post-secondary/university education comprised 70% of the sample, compared to 64% in the general Singaporean population [46], which may limit generalisability of study findings to the wider Singaporean population.

## Conclusion

Children exhibited distinct trajectories of fruit and vegetable intakes from toddlerhood to adolescence. Most children (~ 80%) followed a ‘High stable’ fruit intake or ‘Consistently high’ vegetable intake trajectory. The remaining children followed suboptimal low or declining trajectory of fruit or intake trajectories. Maternal ethnicity, pregnancy diet quality, education, breastfeeding duration and child sex were identified as determinants of suboptimal low or declining fruit or vegetable intake trajectories. Notably, children in the ‘High’ fruit and vegetable intake trajectories had intakes below recommendations, underscoring the urgency of interventions and public health initiatives to increase fruit and vegetable intakes from early life. Such interventions should be initiated from preconception or pregnancy and prioritize Malay mothers, those with lower education, and improve maternal diet quality during pregnancy and promote breastfeeding. Moreover, such interventions should be carried forward until toddlerhood and adolescence, with enhanced strategies for families with boys.

## Supplementary Information

Below is the link to the electronic supplementary material.

Supplementary Material 1

## Data Availability

Data described in the manuscript, code book, and analytic code will be made available upon request pending approval from our study executives.

## Abbreviations

DOHaD: Developmental origins of health and disease
FFQs: Food frequency questionnaires
GBTM: Group based trajectory modelling
GSEM: Generalized structural equation modelling
GUSTO: Growing up in Singapore towards healthy outcomes
HEI-SGP: Healthy eating index for pregnant women in Singapore
WHO: World health organization
