# Supplementary material for: Harnessing generalized structural equation modelling to understand the pathway linking maternal and child factors to fruit and vegetable intake trajectories from toddlerhood to adolescence
Source: Eur J Nutr. 2026 Jan 24;65(1):35. doi: 10.1007/s00394-025-03883-8 (PMC12831672; doi:10.1007/s00394-025-03883-8)
Supplement: Supplementary file 1 — Supplementary Material 1 [file 394_2025_3883_MOESM1_ESM.docx]

Pregnant women at baseline

(n=1450)

Lost to follow-up

n=259

Children born

(n=1201)

Children with no or one dietary intake data at ages 18m, 5y, 7y, 12y were excluded (n=384)

Group-based trajectory analysis

children with ≥2 dietary intake measures at ages 18m, 5y, 7y, 12y were included

n=817

Missing data on child and maternal covariates

(n=136)

Generalised structural equation modelling analysis

n=681

Supplementary Figure 1: Flow chart showing the number of participants included in the analysis assessing the child and maternal determinants of child fruit and vegetable intake trajectories

Supplementary Table 1. Number of fruits and vegetables items at ages 18months, 5, 7 and 12 years

| **18months** | | **5 years** | |
| --- | --- | --- | --- |
| **Fruits** | **Vegetables** | **Fruits** | **Vegetables** |
| Apples and pears | Carrots, pumpkin | Apples and pears | Carrots, pumpkin |
| Bananas | Peas and green beans | Bananas | Peas and green beans |
| Papaya | Sweetcorn | Papaya | Sweetcorn |
| Oranges or citrus fruits | Tomato, red/green peppers | Oranges or citrus fruits | Tomato, red/green peppers |
| Grapes | Dark green leafy vegetables | Grapes | Dark green leafy vegetables |
| Stone fruits | Pale green leafy vegetables | Stone fruits | Pale green leafy vegetables |
| Berries | Broccoli, cauliflower | Tropical fruit (longan, durian, mangosteen, jack fruit) | Broccoli, cauliflower |
| Durian |  | Guava | Stalk vegetables |
| Avocado |  | Dried fruit | Fresh/dried mushroom |
| Raisins |  | Avocado |  |
|  |  | Raisins |  |
| **7 years** | | **12 years** | |
| **Fruits** | **Vegetables** | **Fruits** | **Vegetables** |
| Citrus fruits | Cabbage | Fresh fruits  (apples, orange, bananas, grapes) | Green leafy vegetables |
| Apples and pears | Dark green leafy vegetables |  | Other vegetables (broccoli, carrot, mushroom) |
| Bananas | Broccoli, cauliflower |  |  |
| Grape, berries | Tomato, red/green peppers |  |  |
| Tropical fruits | Carrots |  |  |
| Dried fruits | Peas & green beans |  |  |
|  | Pumpkins |  |  |
|  | Mushroom |  |  |
|  | Okra, eggplant |  |  |
|  | Sweetcorn |  |  |
|  | Stir-fried, stewed, non-coconut curry vegetables |  |  |
|  | Stalk vegetables |  |  |
|  | Gourds |  |  |

Supplementary Table 2. Comparison of baseline characteristics between included and excluded participants

|  | Include | | Exclude | |  |
| --- | --- | --- | --- | --- | --- |
|  | n | % or mean(SD) | n | % or mean(SD) | P-value |
| Child sex | 813 |  | 403 |  | 0.849 |
| Girls | 388 | 52.3% | 213 | 52.8% |  |
| Boys | 425 | 47.7% | 190 | 47.2% |  |
| Child birth weight (kg) | 813 | 3.10(0.48) | 404 | 2.92(0.58) | <0.001 |
| Maternal age (years) | 791 | 31.37(5.11) | 430 | 30.08(4.94) | <0.001 |
| Maternal pregnancy BMI (kg/m^2^) | 773 | 26.18(4.45) | 421 | 26.23(4.39) | 0.868 |
| Maternal pregnancy smoking | 790 |  | 427 |  | 0.09 |
| Non-smoker (No) | 692 | 87.6% | 359 | 84.1% |  |
| Ex-smoker/Current-smoker (Yes) | 98 | 12.4% | 68 | 15.9% |  |
| Maternal ethnicity | 807 |  | 667 |  | 0.008 |
| Chinese | 476 | 53.2% | 355 | 59.0% |  |
| Malay | 205 | 25.0% | 167 | 25.4% |  |
| Indian | 126 | 21.7% | 145 | 15.6% |  |
| Maternal education | 795 |  | 629 |  | 0.023 |
| Secondary/lower | 233 | 29.3% | 226 | 35.9% |  |
| Post-secondary | 283 | 35.6% | 212 | 33.7% |  |
| University | 279 | 35.1% | 191 | 30.4% |  |
| Maternal pregnancy diet quality | 729 | 52.59(13.74) | 398 | 50.54(13.01) | 0.015 |
| Breastfeeding duration | 775 |  | 270 |  |  |
| <6months | 462 | 59.6% | 177 | 65.6% | 0.088 |
| ≥6months | 313 | 40.4% | 93 | 34.4% |  |

Supplementary Table 3. Model fit statistics for selecting fruit and vegetable intake trajectory groups

|  | BIC(N=2510) | BIC(N=817) | Entropy | Proportion of groups (%) |
| --- | --- | --- | --- | --- |
| Fruit intake |  |  |  |  |
| 5(3,3,3,3,3) | -4107.50 | -4093.47 | 0.906 | 1.5%; 2.4%; 75.2%; 4.6%; 16.4% |
| 4(3,3,3,3) | -4157.05 | -4145.83 | 0.917 | 2.3%; 3.6%; 77.7%; 16.4% |
| 3(3,3,3) | -4248.81 | -4240.40 | 0.902 | 16.6%; 79.6%; 3.8% |
| **2(3,3)** | **-4500.35** | **-4494.73** | **0.886** | **16.8%; 83.2%** |
| Vegetable intake |  |  |  |  |
| 5(3,3,3,3,3) | -4716.50 | -4702.46 | 0.875 | 7.5%; 66.4%; 8.4%; 3.9%; 13.9% |
| 4(3,3,3,3) | -4789.76 | -4778.53 | 0.857 | 13.8%; 7.3%; 10.9%; 68.0% |
| **3(3,3,3)** | **-4878.62** | **-4870.20** | **0.881** | **12.0%; 9.0%; 78.8%** |
| 2(3,3) | -4988.76 | -4983.15 | 0.888 | 22.4 %; 77.6% |

Supplementary Table 4. Direct associations between maternal and child factors and fruit and vegetable intake trajectory from toddlerhood to early adolescence.

|  | ‘Low stable to decreasing’  vs ‘High stable’ fruit intake trajectory | | | |  | ‘High decreasing and Low to Stable’ vs ‘Consistently high’ vegetable intake trajectory | | | |
| --- | --- | --- | --- | --- | --- | --- | --- | --- | --- |
|  | OR | 95%CI | | P-value |  | OR | 95%CI | | P-value |
| Child sex (boy vs girl) | 2.32 | 1.51 | 3.68 | <0.001 |  | 1.81 | 1.17 | 2.81 | 0.008 |
| Maternal ethnicity |  |  |  |  |  |  |  |  |  |
| Indian vs Chinese | 0.67 | 0.29 | 1.41 | 0.320 |  | 0.84 | 0.34 | 1.69 | 0.670 |
| Malay vs Chinese | 1.87 | 1.12 | 3.06 | 0.015 |  | 3.49 | 2.14 | 5.60 | <0.001 |
| Maternal education |  |  |  |  |  |  |  |  |  |
| Post secondary vs secondary/lower | 0.97 | 0.63 | 1.67 | 0.910 |  | 0.71 | 0.46 | 1.15 | 0.140 |
| University vs secondary/lower | 0.71 | 0.40 | 1.42 | 0.300 |  | 0.47 | 0.24 | 0.89 | 0.024 |
| Maternal pregnancy diet quality score | 0.98 | 0.97 | 0.99 | 0.029 |  | 0.98 | 0.96 | 0.99 | 0.010 |
| Maternal pregnancy body mass index (kg/m^2^) | 1.02 | 0.97 | 1.07 | 0.450 |  | 1.01 | 0.96 | 1.05 | 0.730 |
| Maternal pregnancy smoking (Yes vs No) | 1.25 | 0.70 | 2.23 | 0.450 |  | 1.21 | 0.70 | 2.11 | 0.490 |
| Breastfeeding duration (≥6 vs 6 months) | 0.57 | 0.36 | 0.92 | 0.019 |  | 0.65 | 0.40 | 1.04 | 0.077 |

Supplementary Table 5. Direct associations between maternal and child factors.

|  | OR | 95%CI | | P-value |
| --- | --- | --- | --- | --- |
| Maternal education (post-secondary vs secondary/lower) | | | | |
| Maternal ethnicity (Indian vs Chinese) | 1.15 | 0.72 | 1.89 | 0.566 |
| Maternal ethnicity (Malay vs Chinese) | 2.10 | 1.47 | 3.04 | <0.001 |
| Maternal education (university vs secondary/lower) | | | | |
| Maternal ethnicity (Indian vs Chinese) | 1.19 | 0.75 | 1.78 | 0.423 |
| Maternal ethnicity (Malay vs Chinese) | 0.07 | 0.03 | 0.13 | <0.001 |
| Breastfeeding duration (≥6 vs 6 months) |  |  |  |  |
| Maternal education (post-secondary vs secondary/lower) | 1.45 | 0.95 | 2.35 | 0.110 |
| Maternal education (university vs secondary/lower) | 4.52 | 2.96 | 7.18 | <0.001 |
| Maternal BMI (kg/m^2^) | 0.95 | 0.91 | 0.98 | 0.005 |
| Maternal diet quality | 1.02 | 1.004 | 1.03 | 0.010 |
| Maternal smoking (ex-smoker/current smoker vs non-smoker) | 0.38 | 0.18 | 0.75 | 0.008 |
| Maternal smoking (ex-smoker/current smoker vs non-smoker) |  |  |  |  |
| Maternal ethnicity (Indian vs Chinese) | 0.66 | 0.14 | 1.84 | 0.523 |
| Maternal ethnicity (Malay vs Chinese) | 3.40 | 1.90 | 6.11 | <0.001 |
| Maternal education (post-secondary vs secondary/lower) | 0.65 | 0.38 | 1.10 | 0.113 |
| Maternal education (university vs secondary/lower) | 0.05 | 0.0000 | 0.19 | 0.434 |
|  | β | 95%CI | | P-value |
| Maternal BMI (kg/m^2^) |  |  |  |  |
| Maternal ethnicity (Indian vs Chinese) | 3.05 | 2.20 | 3.97 | <0.001 |
| Maternal ethnicity (Malay vs Chinese) | 3.00 | 2.22 | 4.03 | <0.001 |
| Maternal education (post-secondary vs secondary/lower) | -0.25 | -1.13 | 0.69 | 0.590 |
| Maternal education (university vs secondary/lower) | -1.21 | -2.04 | -0.43 | 0.003 |
| Maternal diet quality |  |  |  |  |
| Maternal ethnicity (Indian vs Chinese) | 0.50 | -2.38 | 3.16 | 0.725 |
| Maternal ethnicity (Malay vs Chinese) | -4.85 | -7.20 | -2.10 | <0.001 |
| Maternal education (post-secondary vs secondary/lower) | 0.32 | -2.20 | 2.73 | 0.800 |
| Maternal education (university vs secondary/lower) | 4.97 | 2.14 | 7.60 | <0.001 |
| Maternal smoking (ex-smoker/current smoker vs non-smoker) | -3.20 | -6.47 | 0.12 | 0.057 |
